# Variation in gene expression along an elevation gradient of *Rhododendron sanguineum* var. *haemaleum* assessed in a comparative transcriptomic analysis

**DOI:** 10.3389/fpls.2023.1133065

**Published:** 2023-03-21

**Authors:** Lin-Jiang Ye, Michael Möller, Ya-Huang Luo, Jia-Yun Zou, Wei Zheng, Jie Liu, De-Zhu Li, Lian-Ming Gao

**Affiliations:** ^1^ CAS Key Laboratory for Plant Diversity and Biogeography of East Asia, Kunming Institute of Botany, Chinese Academy of Sciences, Kunming, Yunnan, China; ^2^ Key Laboratory of Plant Resources and Biodiversity of Jiangxi Province, Jingdezhen University, Jingdezhen, Jiangxi, China; ^3^ Germplasm Bank of Wild Species, Kunming Institute of Botany, Chinese Academy of Sciences, Kunming, Yunnan, China; ^4^ Royal Botanic Garden Edinburgh, Edinburgh, Scotland, United Kingdom; ^5^ Lijiang Forest Biodiversity National Observation and Research Station, Kunming Institute of Botany, Chinese Academy of Sciences, Lijiang, Yunnan, China; ^6^ University of Chinese Academy of Sciences, Beijing, China

**Keywords:** *Rhododendron*, elevational gradients, evolutionary adaptation, RNA-seq, organ-specific profile, alpine ecosystem

## Abstract

Selection along environmental gradients may play a vital role in driving adaptive evolution. Nevertheless, genomic variation and genetic adaptation along environmental clines remains largely unknown in plants in alpine ecosystems. To close this knowledge gap, we assayed transcriptomic profiles of late flower bud and early leaf bud of *Rhododendron sanguineum* var. *haemaleum* from four different elevational belts between 3,000 m and 3,800 m in the Gaoligong Mountains. By comparing differences in gene expression of these samples, a gene co-expression network (WGCNA) was constructed to identify candidate genes related to elevation. We found that the overall gene expression patterns are organ-specific for the flower and leaf. Differentially expressed unigenes were identified in these organs. In flowers, these were mainly related to terpenoid metabolism (*RsHMGR*, *RsTPS*), while in leaves mainly related to anthocyanin biosynthesis (*RsCHS*, *RsF3’5’H*). Terpenoids are the main components of flower scent (fragrance) likely attracting insects for pollination. In response to fewer pollinators at higher elevation zone, it seems relatively less scent is produced in flower organs to reduce energy consumption. Secondary metabolites in leaves such as anthocyanins determine the plants’ alternative adaptive strategy to extreme environments, such as selective pressures of insect herbivory from environmental changes and substrate competition in biosynthesis pathways at high elevations. Our findings indicated that the gene expression profiles generated from flower and leaf organs showed parallel expression shifts but with different functionality, suggesting the existence of flexibility in response strategies of plants exposed to heterogeneous environments across elevational gradients. The genes identified here are likely to be involved in the adaptation of the plants to these varying mountainous environments. This study thus contributes to our understanding of the molecular mechanisms of adaptation in response to environmental change.

## Introduction

Environmental adaptation and its driving mechanisms in speciation have always been one of the most challenging and unresolved key research aspects in ecology and evolutionary biology (Nelson et al., 2007; Anderson et al., 2011). Adaptation is an evolutionary process in which a species inherits its genetic material by changing its shape or adjusting its survival strategy in order to better adapt to its living environment (Mayr, 1982). In mountain ecosystems, variances in environmental factors due to elevational gradients are considered to be eminently more significant than environmental changes over horizontal gradients. Elevational gradients influence environmental variables, causing major shifts in biotic and abiotic factors within a relatively short geographic distance (Körner, 2007; Liu et al., 2022b), which provides a natural laboratory for the study of environmental adaptation. Compared with low-elevation areas, species inhabiting high-elevation environments are subjected to multiple selective pressure, including low temperature and oxygen levels, high intensity of ultraviolet radiation and strong seasonality (Qiu, 2008; Zhang et al., 2019). Hence, understanding the molecular basis of how species adapt to different environments along elevation gradients can make a significant contribution to our knowledge of adaptation.

Recently, demonstration of how wild species adapt to their local environments has been made easier with the developing of genomic approaches based on next-generation sequencing (NGS) technologies. Especially the transcriptomic analyses have become an effective tool for further research on gene expression, gene regulation, and species adaptive evolution, greatly facilitating eco-evolutionary research on non-model organisms that lack reference genome sequence information (Stapley et al., 2010; Ekblom and Galindo, 2011; Mutz et al., 2013). To date, several studies on the adaptation to divergent alpine environments have been carried out on animals (e.g. Sun et al., 2018; Hao et al., 2019; Qu et al., 2020; Ma et al., 2022a). For plants, a few recent studies have reported the genetic architectures and evolutionary processes driving their adaption along elevational gradients (e.g. Ma et al., 2015; Bohutinska et al., 2021; Liu et al., 2022b). However, intraspecific local adaptation of alpine plants across the extreme elevational gradients existing in the Hengduan Mountains is very scarce, in particular for woody species occurring at an elevation greater than 3,000 m.

*Rhododendron* (Ericaceae) is a species-rich and ecologically important genus with over 1,000 species globally (Chamberlain et al., 1996), and approximately 590 species occurring in China with a diversification center in the Himalaya-Hengduan Mountains (HHM) (Yan et al., 2015; Fu et al., 2022). *Rhododendron* underwent rapid adaptive radiation after the Late Miocene in HHM (Mo et al., 2022; Ma et al., 2022b), which led to many sympatrically distributed species in this region (Zou et al., 2021). In addition, *Rhododendron* can inhabit diverse environments between 1,000 m up to nearly 5,000 m and represent an important component of these heterogeneous ecosystems (Shrestha et al., 2018; Liu et al., 2022b), and environmental heterogeneity was proven to have the highest effects on species diversity in the genus (Shrestha et al., 2018; Xia et al., 2022). Despite increasing awareness of their ecological importance, the molecular mechanisms of *Rhododendron* species that underpin adaptation to environmental changes along elevation gradients are poorly studied.

Recently, high-throughput RNA Sequencing (RNA-seq) has been widely applied to explore plant responses to high-elevation environments. For example, a combination of transcriptomic and metabolomic analyses revealed that differences in gene expression, evolutionary adaptation rate and metabolites changes are involved in the adaptation of four differently flower-colored *Rhododendron* species to heterogeneous environments across elevational gradients (Liu et al., 2022b). *Rhododendron sanguineum* forms a highly color polymorphic complex group including six varieties and is typically located at elevations over 3,000 m, which is associated with snow cover (Fang et al., 2005; Ye et al., 2021). The plants are shrubs that typically grow 30 cm–150 cm in height and are exposed in the open (Fang et al., 2005). Our previous comparative transcriptomics study has clarified the molecular mechanisms of flower color divergence of three sympatrically occurring varieties in this complex (Ye et al., 2021). However, how the *R. sanguineum* complex has adapted and evolved to the highly heterogenous environments along elevational gradients above 3,000 m remains unclear. The adaptation of plants of one variety, *R. sanguineum* var. *haemaleum*, to their specific habitat has likely contributed to its intraspecific diversification, and these plants should be an ideal system for the study of ecological adaptation in a heterogeneous environment.

The present study was designed to reveal adaptation mechanisms at the gene expression level of an alpine *Rhododendron sanguineum* variety across elevational gradients where the plants occur. We generated gene expression profiles using RNA-seq data from two major organs including late flower and early leaf buds of *R*. *sanguineum* var. *haemaleum* growing at four different elevation zones between 3,000 m and 3,800 m in the Gaoligong Mountains. A comparative transcriptome analysis of the organs from different elevations was performed to determine their gene expression patterns to identify differentially expressed unigenes related to elevation. We then annotated these genes to characterize their likely functions to reveal the response strategies of different organs to heterogenous environments underlying elevation adaptation. This study will provide novel insights into the genetic mechanisms of alpine plant species in response to heterogeneous environments caused by elevation and lay a foundation for further explorations of the genetic changes underlying high elevational adaptation among rhododendrons and other alpine species. In addition, to the best of our knowledge, it is the first comparative transcriptomic study on the adaptation mechanism of an intraspecific alpine woody plant to elevational gradients under field conditions.

## Materials and methods

### Plant sampling

**Figure 1:**
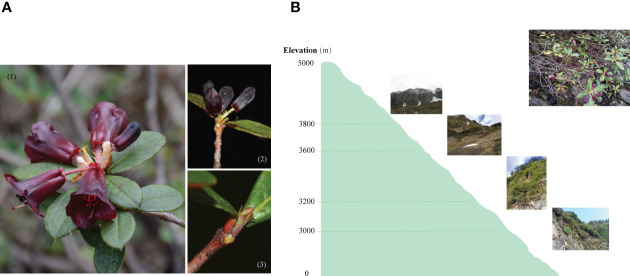
Morphological characters, sampling organs **(A)**, schematic sampling map and wild habitats **(B)** of *R*. *sanguineum* var. *haemaleum*. Morphology of open flowers and fully expaned leaves (1), sampled stage for entire late flower buds (2), and entire early leaf buds (3).

In June 2018, samples of *R*. *sanguineum* var. *haemaleum* for transcriptome sequencing were collected between 11:00am and 1:30pm within two successive days from four sites along an elevational gradient (**Figure 1**) in the northern Gaoligong Mountains (N 27°49’53”, E 98°27’03” - N 28°05’13”, E 98°45’31”) which is located in southern of Hengduan Mountains (Liu et al., 2022a). Three individuals were sampled across a small range of 20 m^2^ from each site. The entire late flower bud and entire early leaf bud were sampled from each individual, immediately frozen in liquid nitrogen in the wild, and preserved in a -80°C ultra-low temperature freezer prior to processing for total RNA isolation. Vouchers of each individual plant sampled were collected and deposited in the Herbarium of Kunming Institute of Botany (KUN), Chinese Academy of Sciences.

### RNA extraction and transcriptome sequencing

Total RNA was extracted and purified separately from each organ using a Spectrum TM Plant Total RNA Kit (STRN250, Sigma) according to the manufacturer’s protocols. The RNA purity and quality were assessed with a Nanodrop 2000 spectrophotometer (Thermo Fisher Scientific, Waltham, MA, USA). RNA integrity was evaluated through agarose gel electrophoresis. Three biological replicates (from three plants) of each flower bud and leaf bud material were included for each elevational site. A single cDNA library was separately constructed for each flower bud and each leaf bud of each individual from each site, and subsequently sequenced on an Illumina HiSeq X Ten sequencing platform (San Diego, CA, USA), generating approximate 6 Gb paired-end reads (2 × 150 bp) for each sample. Both cDNA library preparation and Illumina sequencing were carried out by Novogene Bioinformatics Technology Co., Ltd. (Beijing, China).

### Data processing, *De novo* assembly and mapping

We first cleaned the raw sequence reads by using Trimmomatic v. 0.38 (Bolger et al., 2014) with default settings to remove low-quality reads. We further evaluated the quality of the remaining reads using FastQC (Andrews, 2010). All subsequent analyses were based on these cleaned reads. A reference transcriptome was generated by *de novo* assembly of the combined clean reads (leaf buds, flower buds, in three replicates from each gradient) performed using Trinity v. 2.6.5 (Haas et al., 2013) with default parameters. Assembly statistics were obtained using the TrinityStats.pl script in the Trinity package. The longest transcripts were considered as the non-redundant unigenes. We also used HISAT2 v. 2.1.0 (Kim et al., 2019) to assess assembly quality, by mapping reads back to the assembled transcripts to count the overall alignment rates.

### Assessment of completeness and gene functional annotation

To lower the redundancy in the dataset, low-coverage artifacts or redundancies were removed using the CD-HIT-EST v. 4.7.0 (Fu et al., 2012) with setting word length to 10 and an identity threshold of 90%. Downstream analyses were performed on the final filtered transcripts. To determine the transcriptome completeness of the assembly, Benchmarking Universal Single-Copy Orthologs tools (BUSCO, v. 4.0.6) (Simao et al., 2015) were used to obtain the percentage of single-copy orthologs represented in the embryophyte database (odb10, 1,614 single-copy orthologues) and also to evaluate the completeness of transcript assemblies. To annotate the assembled unigenes, we downloaded a protein reference of *R. delavayi*, a species closely related to *R. sanguineum* from the whole genome sequencing project deposited in GigaDB (Zhang et al., 2017). Open reading frames (ORFs) were first predicted from each filtered assembled transcript using TransDecoder v. 5.5.0 (Haas et al., 2013) with a minimum length of 100 amino acids, and the predicted ORFs were scanned to find homology profiles with a cut-off e-value of 1e-10 against the reference protein database. Unigenes and predicted protein sequences were used as queries to search protein databases using the BLASTP v. 2.5.0, setting the e-value cutoff to 1e-10. Queries were performed against the NCBI non-redundant (NR) and UniProtKB/Swiss-Prot databases. We also performed additional functional annotations with DIAMOND (Buchfink et al., 2015) hits against the eggNOG database (Huerta-Cepas et al., 2017), which summarized available functional information from the different proteins databases, including GO (Gene Ontology), COGs/KOGs (Clusters of Orthologous Groups, containing both prokaryotic and eukaryotic clusters), Pfam (Protein families) and KEGG (Kyoto Encyclopedia of Genes and Genomes) (Hernández-Plaza et al., 2023). The best hit was used as the final annotation.

### Transcript abundance and differential expression analyses

To quantify transcript abundance, we applied the alignment-based methods by mapping all of the cleaned reads from the flower bud samples separately for each biological replicate back to the non-redundant unigenes of the assembled reference transcripts using RSEM v. 1.3.1 (Li and Dewey, 2011) and Bowtie2 v. 2.3.5 (Langmead and Salzberg, 2012) for alignment. When the transcript abundance for each biological replicate flower bud sample had been obtained, we generated a gene expression matrix that was constructed from a matrix of read count with a Trinity script. The differentially expressed unigenes (DEUs) analysis were performed with the DESeq2 package (Love et al., 2014) among the four elevation gradients. Samples from the three high elevations were separately compared to those harvested at lower elevation (3,000 m) to construct comparison groups. DEUs were considered those with false discovery rate (FDR) adjusted *p* values ≤ 0.05 and absolute values of log2 (fold change) ≥ 1. To compare gene expression values across four populations at different elevations, we used the trimmed mean of M-values normalization (TMM), as implemented in the *R* package edgeR. All downstream analyses were implemented based on the normalized expression data matrix (TMM normalization). The functional enrichment analyses of DEUs from each comparison were further processed as described previously (Ye et al., 2021), including GO (Gene Ontology) and KEGG (Kyoto Encyclopedia of Genes and Genomes). GO terms and metabolic pathways with *p* values ≤ 0.05 were considered significantly enriched by DEUs.

### Construction of weighted gene co-expression network

WGCNA (Weighted gene co-expression network analysis) is an analytical method to explore the relationship between modules and concerned phenotypes and to mine key genes in co-expression networks (Langfelder and Horvath, 2008). To further identify the relevant regulatory gene modules related to elevation, a gene co-expression network was constructed on flower and leaf bud organs, respectively, using the WGCNA package in *R* (Langfelder and Horvath, 2008). This pipeline included principally three steps. Firstly, a hierarchical cluster analysis was carried out after removing low correlation genes, or those with low expression levels using the hclust function. Secondly, to meet the prerequisite of scale-free network distribution, the soft-power threshold *β* was determined by the function “sft$powerEstimate” to create an adjacency matrix. To better assess the correlation-based association among gene expression patterns, the adjacency matrix was further converted to a topological overlap matrix (TOM), and a gene connection network was constructed. Finally, gene modules were identified and clustered by a dynamic tree cut method based on the eigengenes (ME) of each module, and the modules with closer distances were merged to obtain the appropriate modules. For the two different organs, the threshold to merge similar modules (mergeCutHeight) and minimal gene module size (minModuleSize) were all set to 0.2 and 100, respectively, except the expression split threshold (deepSplit, flower bud was 2 and leaf bud was 1).

### Identification and enrichment analysis of target gene modules

To further investigate the gene modules associated with different environmental conditions, the correlation coefficients between module eigengenes and different altitudes were calculated and statistically tested using Pearson correlation analyses. Modules with absolute values of correlation coefficients > 0.60 (*P* < 0.01) were considered as altitude-relevant modules: the larger the absolute values of correlation coefficient, the higher the correlation between the module and altitude. Functional enrichment analyses of DEUs in altitude-related modules were carried out by using the AnnotationForge and clusterProfiler package in *R* (Yu et al., 2012; Carlson and Pages, 2018).

## Results

### Illumina sequencing, *De novo* assembly and quality assessment

The transcriptome data of 24 cDNA libraries originating from the two organs (late flower buds and early leaf buds) for three individuals from each of the four elevational samples were obtained in this study. After trimming adapter sequences and removing low-quality sequences, we generated 40,622,598 – 59,403,462 sequence reads with the Q30 percentage of 89.22% – 93.82% and the GC percentage of 46.35% – 48.84% (**Supplementary Table S1**). A total of ~174.4 Gb clean data was generated after merging all reads of the 24 libraries. The Q30 (99.9% base call accuracy), GC-content of the merged data were 92.60% and 43.52%, respectively (**Table 1**). All of the clean reads were assembled into transcripts using the *de novo* assembly tool. In total, 335,121 transcripts were identified with the average length of 1,156 bp and N50 length of 1,707 bp. We selected the longest transcript as the representative for each cluster. All the transcripts were further filtered to obtain 162,995 unigenes with the average length of 907 bp, N50 length of 1,201 bp and GC-content of 42.02% (**Table 1**). The majority of the assembled unigenes were in the ranges of 300–2,000 bp and 14,698 (9.02%) were over 2,000 bp (**Table 1**, **Supplementary Figure S1B**). Assessment of the transcriptome completeness identified 1,543 (95.60%) complete and fragmented BUSCOs based on the 1,614 conserved BUSCO embryophyte orthologs (**Supplementary Figure S1A**). The reads of the 24 cDNA libraries were aligned by mapping the reads back to the assembled reference transcript, with mapping rates ranging from 93.85% to 96.07% (**Supplementary Table S1**). These indicated that our transcriptomes were well assembled, and were of good quality.

**Table 1:** Summary of sequencing data, assembly and function annotation of the *R. sanguineum* var. *haemaleum* transcriptome.

Original data	Values	Percentage (%)
Total clean reads	1,162,569,464	**-**
Total length (Gb)	174.4	**-**
Q30 percentage	92.60%	**-**
GC percentage	43.52%	**-**
Assembly		
Total number of unigenes	162,995	**-**
N50 (bp)	1201	**-**
Average length (bp)	907	**-**
Unigenes (>1 kb)	41,531	**-**
Unigenes (>2 kb)	14,698	**-**
GC content (%)	42.02%	**-**
TransDecoder		
Total number of proteins	65,147	**-**
Filtered proteins	64,233	**-**
Annotation		
BLASTx against NR	49,776	77.49
BLASTx against Uniprot	29,694	46.23
BLASTx against COG	42,709	66.49
BLASTx against Pfam	44,380	69.09
All annotated transcripts	53,334	83.03
Transcripts matching all four databases	27,802	43.28
Functional classification and pathway mapping		
Annotated with Gene Ontology (GO) terms	22,454	34.96
Annotations against KEGG	15,553	24.21

### Functional annotation of the transcriptome

To determine putative functions of the identified unigenes of *R*. *sanguineum* var. *haemaleum*, partial and complete open reading frames (ORFs) were predicted using the TransDecoder software, and CD-HIT-EST was used to cluster protein coding transcripts to obtain the non-redundant proteins. Finally, a total of 64,233 coding proteins were translated from the filtered assembly (**Table 1**). Similarity searches for each unigene against public databases, including National Center for Biotechnology Information non-redundant (NR) protein database, UniProt, COG and Pfam found 49,7769 (77.49%), 29,694 (46.23%), 42,709 (66.49%), 44,380 (69.09%) matches, respectively. Taken together, 53,334 (83.03% of the total) of the unigenes showed a match in at least one of these searchable databases, and 27,802 (43.28%) showed significant matches in all of the four databases (**Table 1**; **Supplementary Figure S1C**). Furthermore, 22,454 (34.96%) unigenes were classified into different GO functional groups. The most abundant GO subcategories for biological processes, molecular functions, and cellular components were cellular process 17,635 (27.45%), catalytic activity 10,098 (15.72%) and cell 19,384 (30.18%), respectively (**Table 1**; **Supplementary Figure S2A**). We assigned 15,553 (24.21%) unigenes to 405 KEGG metabolic pathways (**Table 1**), which represents a valuable resource for investigating specific processes, functions and pathways. Details regarding the KEGG annotation can be found in **Supplementary Figure S2B**.

### Differentially expressed unigenes and functional enrichment

**Figure 2:**
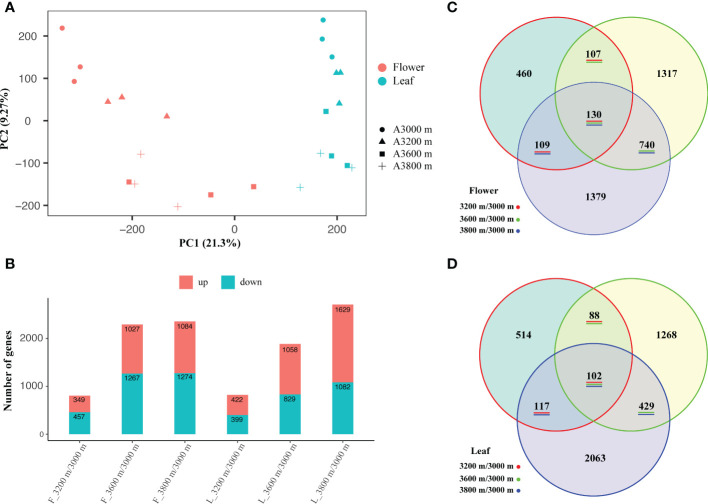
Principal component analysis (PCA) of 24 individuals of the *R*. *sanguineum* var. *haemaleum* **(A)**, and the number statistics **(B)** and Venn diagrams **(C, D)** of differentially expressed unigenes (DEUs) among three different comparisons in late flower and early leaf bud, respectively.

All expression analyses were performed separately for the three biological replicates of the flower and leaf organs. A gene expression matrix with 24 columns and 162,995 lines were generated. Each column represented a sample and each line corresponded to the expression of a unigene. The gene expression density and gene expression distribution of 24 individual libraries in late flower buds and early leaf buds can be found in **Supplementary Figure S3**. PCA analysis and correlation matrix showed a good correlation between the replicate sets for each elevation of the 24 individual libraries, each elevation and different organ clustered together, showing a population-specific and organ-specific pattern (**Figure 2A**), which indicated that the RNA-seq libraries were reliable.

Based on the expression profiles at different elevational sites, differentially expressed unigenes (DEUs) were identified by comparing them to the lowest elevation (3,000 m). A total of 2,358 DEUs (up-regulated: 1,084, down-regulated: 1,274) were obtained in the comparison 3,800 m *vs*. 3,000 m, followed by 3,600 m *vs*. 3,000 m (2,294, up-regulated: 1,027, down-regulated: 1,267) and 3,200 m *vs*. 3,000 m (806, up-regulated: 349, down-regulated: 457) in late flower bud, while 2,711 DEUs (up-regulated: 1,629, down-regulated: 1,082), 1,887 DEUs (up-regulated: 1,058, down-regulated: 829), and 821 DEUs (up-regulated: 422, down-regulated: 399) were screened in early leaf bud, respectively (**Figure 2B**). These graphs indicated that the number of DEUs increased with increasing elevation span in each comparison group of the two organs. In total, we generated 130 DEUs in three comparisons of late flower bud (**Figure 2C**) and 102 DEUs in early leaf bud (**Figure 2D**). Details of the DEUs pattern are shown in **Supplementary Figure S4**.

The GO and KEGG functional enrichment analyses of the shared DEUs in the three comparisons showed that the GO enrichment terms of the DEUs were classified into three categories of gene ontologies: biological processes (BP), cellular components (CC) and molecular functions (MF). As shown in **Supplementary Figure S5A**, the most significantly enriched GO terms in the two different organs (late flower bud, early leaf bud) were sesquiterpene metabolic processes (GO:0051761) and negative regulation of leaf senescence (GO:1900056), respectively, while the KEGG enrichment analysis showed that the DEUs were significantly enriched in sesquiterpenoid and triterpenoid biosynthesis (ko00909), fat digestion and absorption (ko04975), photosynthesis-antenna proteins (ko00196) and tropane, piperidine and pyridine alkaloid biosynthesis (ko00960) (**Supplementary Figure S5B**). The genes enriched in these GO terms and KEGG pathways may be closely related to the species’ elevational adaptability.

### Weighted gene co-expression network construction and module identification

**Figure 3:**
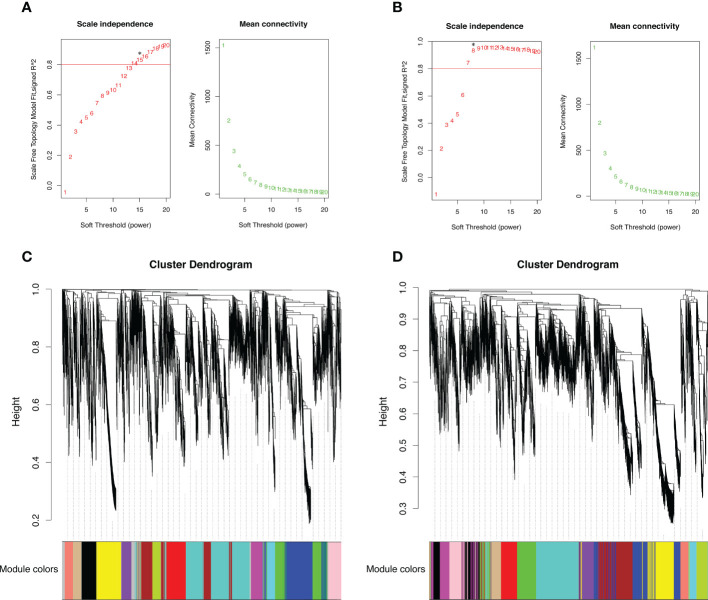
Plot of scale free topology and mean connectivity in regard to soft-thresholding power and Clustering dendrogram showing ortholog expression pattern for samples from **(A, C)** late flower bud and **(B, D)** early leaf buds. Red line indicates an R^2^ cut-off of 0.8. Asterisk indicates the soft threshold power chosen for module detection. Each colored bar below represents each module.

In the WGCNA analysis, to further explore the specific genes that were highly associated with high-elevation adaptation in different organs, separately performed based on the DEUs from late flower buds and early leaf buds, we independently identified 4,242 and 4,581 DEUs among the three comparisons in the two organs. Based on the scale-free topology criterion (scale-free *R* ^2^ of 0.80 and mean connectivity close to 0), the soft-power threshold of *β =*15 and 8 were selected to generate a hierarchical tree of flower and leaf bud, respectively (**Figures 3A, B**). The gene modules were detected based on the topological overlap matrix (TOM), and genes from the two organs were all assigned into 15 distinct modules based on the similarity of their expression patterns. The numbers of the genes in each module varied greatly from 124 to 880 in late flower bud, and 127 to 759 in early leaf bud, regardless of those unclassified genes in grey modules (**Figures 3C, D**).

### Analysis of elevation related genes in the target modules

**Figure 4:**
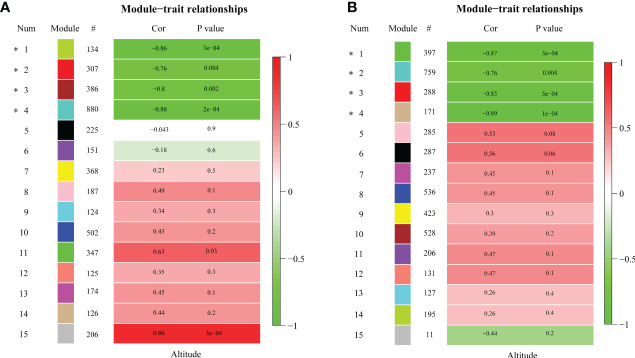
Pearson’s correlation coefficient (Cor) and corresponding significance level (*P* value) between the expression of genes in each module and the sample altitude from **(A)** late flower buds and **(B)** early leaf buds. “#” indicates the number of genes in each module. Asterisk indicates altitude-related module. The color bar to the right of each figure indicates the strength of correlation coefficient.

To characterize the key modules associated with elevation in *R. sanguineum* var. *haemaleum*, we calculated the correlation coefficients between the module and elevation. Modules with the module-trait relationships |r| > 0.60 and *p* < 0.01 were selected as the key ones that were significantly associated with elevation. Among the 15 gene co-expression modules, four specific modules were obtained in different organs of flowers and leaves, and all of them were significantly negatively correlated with altitude (r < 0), implying that DEUs in these modules may be positively expressed at lower elevations in the flower and leaf organs of *R. sanguineum* var. *haemaleum*. The turquoise module was significantly correlated with elevation (*r* = -0.88, *p* = 2e-04) (**Figure 4**), followed by the green module (*r* = -0.86, *p* = 3e-04), brown module (*r* = -0.8, *p* = 0.002) and red module (*r* = -0.76, *p* = 0.004), with 880, 134, 386 and 307 DEUs assigned respectively, in late flower bud. While, in early leaf bud, the tan module (*r* = -0.89, *p* = 1e-04) with 171 DEUs, the green module (*r* = -0.87, *p* = 3e-04) with 397 DEUs, the red module (*r* = -0.85, *p* = 5e-04) with 288 DEUs, and the turquoise module (*r* = -0.76, *p* = 0.004) with 759 DEUs were significantly correlated (**Figure 4**).

**Figure 5:**
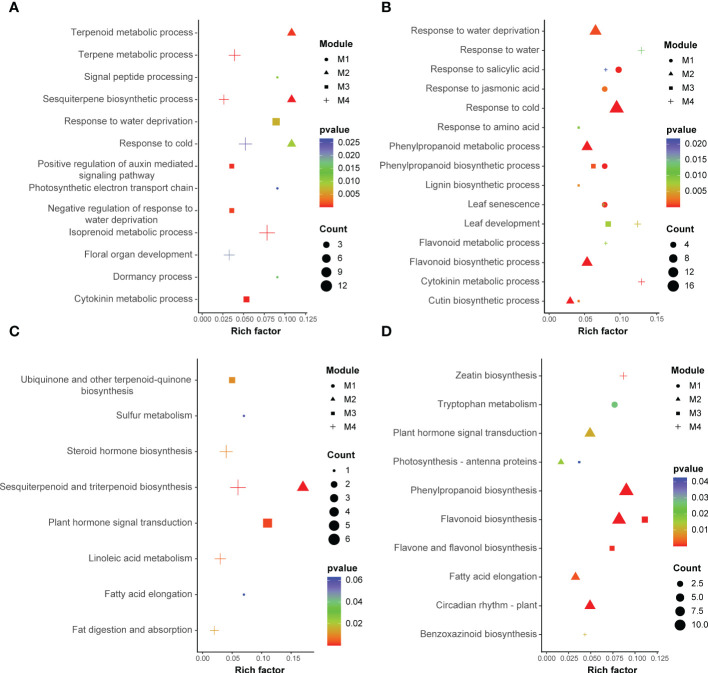
Results of GO enrichment **(A, B)** and KEGG enrichment **(C, D)** of significantly expressed unigenes in four different modules identified from late flower buds **(A, C)** and early leaf buds **(B, D)**, respectively. “M1”, “M2”, “M3” and “M4” refer to the modules that were significantly correlated with altitude.

In the GO and KEGG analyses, conducted to investigate the biological function of the genes in each altitude-related module, the former indicated terpenoid metabolic processes (GO:0006721), response to water deprivation (GO:0009414), plant hormone signal transduction (ko04075) and sesquiterpenoid and triterpenoid biosynthesis (ko00909) as the most significantly enriched functional annotations in late flower buds, and phenylpropanoid biosynthetic processes (GO:0009699), leaf senescence (GO:0010150), flavonoid biosynthesis (ko00941) and photosynthesis-antenna proteins (ko00196) as the most significantly enriched terms/metabolic pathways in early leaf buds. These were the key enrichment objects for the subsequent analysis (**Figure 5**). Additionally, there were some GO categories or metabolic pathways that were shared in the two organs, while the organ-specific enrichment types were the most striking ones worthy of further analysis, such as sesquiterpene biosynthetic processes (GO:0051762) that occur only in floral organ, and flavonoid biosynthesis (ko00941) unique to leaf organ.

**Figure 6:**
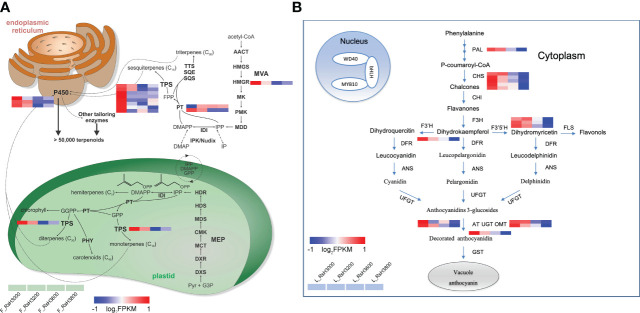
Schematic overview of major terpenoid **(A)** (modified from Karunanithi and Zerbe, 2019) and flavonoid **(B)** biosynthetic pathway identified in the organ of late flower buds and early leaf buds, respectively. Altitudes (here RsH3000-RsH3800, from left to right in each heatmap panel) are indicated in the legend. Gene expression profile (in normalized FPKMs) at different elevations are presented in a heatmap alongside the gene names. The bar represents the expression level of each gene. Low to high expression is indicated by a change in color from blue to red, respectively.

Based on the result of the GO and KEGG enrichment of DEUs, we confirmed the functional annotations of all candidate genes (**Table 2**). The sample expression patterns were clustered and visualized in a heatmap to clearly understand the expression of all candidate genes from the two organs (**Supplementary Figure S6**). In both organs, most gene expression patterns were negatively correlated with altitude, in particular genes related to terpene metabolism in the late flower bud (*RsHMGR*, *RsPT*, *RsTPS*) and anthocyanin metabolism (*RsCHS*, *RsF3’5’H*, *RsOMT*) in the early leaf bud (**Table 2**). The genes in the terpenoid biosynthesis pathway identified in late flower bud and in the anthocyanin metabolism pathway of early leaf bud were found highly expressed at low elevations and decreased with increasing elevation (**Figure 6**). Additionally, some genes had multiple homologous copies, with the largest number of 8 and 5 in *RsTPS* and *RsCHS* respectively (**Table 2**), which to a certain extent indicated the importance of these two genes in their respective pathways. However, genes related to each node were not enriched across the entire metabolic pathway (**Figure 6**).

**Table 2:** Information of the candidate genes associated with elevation, involved in the biosynthetic pathway of late flower bud and early leaf buds.

Pathway	Enzyme	Uniprot ID	Annotation	Unigenes
Terpenoid biosynthesis	HMGR	A0A0A1C3I2	3-hydroxy-3-methylglutaryl coenzyme A reductase	DN1574_c1_g1
	TPS1	Q6Q3H3	germacrene D synthase	DN13962_c0_g1, DN14863_c0_g1, DN24679_c0_g1, DN1003_c0_g1, DN1331_c0_g1, DN13379_c0_g1, DN17954_c0_g1, DN51811_c1_g2
	TPS3	U3KYL2	drimenol synthase	DN6858_c0_g2
	PT	Q8VYB7	prenyl transferase	DN1150_c0_g1, DN1985_c0_g1
	CYP71	Q42716	Cytochrome P450 71A8	DN36882_c0_g1, DN4539_c0_g1
	CYP736	A0A068Q6L2	Cytochrome P450 736A117	DN39393_c0_g1
Flavonoid biosynthesis	PLA	P45726	Phenylalanine ammonia-lyase	DN3288_c1_g1
	CHS	P48386	Chalcone synthase	DN1880_c2_g1, DN31591_c0_g1, DN17261_c0_g2, DN1880_c17_g1, DN33271_c0_g1
	F3’5’H	P48418	Flavonoid 3’,5’-hydroxylase	DN14411_c0_g1, DN346_c8_g1, DN2888_c0_g1
	F3’H	Q9SBQ9	Flavonoid 3’-monooxygenase	DN448_c0_g1
	AT	A0A2P1GIW7	acetyltransferase	DN2772_c0_g4, DN414_c1_g2
	OMT	Q43047	Caffeic acid 3-O-methyltransferase	DN4372_c7_g1, DN2109_c1_g3
	UGT	V5LLZ9	Gallate 1-beta-glucosyltransferase	DN4870_c0_g1
	CYP73	Q43054	Trans-cinnamate 4-monooxygenase	DN8935_c0_g1
	CYP84	Q42600	Cytochrome P450 84A1	DN6826_c0_g1
	CYP98	O48922	Cytochrome P450 98A2	DN4304_c0_g1

## Discussion

### Gene expression profiles across organs of *R. sanguineum* var. *haemaleum*

In this study, we carried out a transcriptome expression profiling analysis in flower and leaf bud sampled along an elevational gradient of *R. sanguineum* var. *haemaleum*. The results showed a organ-specific expression profile pattern (**Figure 2A**), which has been proven in other studies (Ye and Varner, 1991; Leung et al., 2014; Tu et al., 2021). Our results were similar to those of Hao et al. (2019) whose work based on comparative transcriptomics of three closely related bird species at different elevations. The sample replicates, *i.e.*, sampled from three individual plants at each elevation, clustered together but separately for elevation (**Figure 2A**; **Supplementary Figure S6A**), indicating that plants growing in the same habitat have a more similar expression profile, while differing from those growing at different elevations. Environmental conditions vary at different elevations, and factors, such as temperature, moisture, light quality, and oxygen, will likely to be involved in determining gene expression levels (Gibson, 2008; Liu et al., 2022b). In addition, by comparing the high and low elevational samples of different organs, *i.e.*, late flower buds and early leaf buds, the number of significantly DEUs in their respective comparisons showed a positive correlation with the magnitude of the elevational distance, *i.e.*, the greater the elevation span, the greater the number of differentially expressed unigenes (**Figure 2B**). This is consistent with other studies on *Potentilla* L. (Ma et al., 2015), *Bos* L. (Ma et al., 2022a) and other species of *Rhododendron* (Liu et al., 2022b). In this sense, one could hypothesize that along a mountain cline, with increasing elevation, the stresses caused by environmental factors become more severe, and as adaptation of species to these changing environments additional genes are activated, also known as environmental adaptation (Sun et al., 2018; Wang et al., 2021b). Therefore, elevation difference may be the most direct factor in affecting gene expression, and it also indicated that there were no differences between the selected organs (late flower and early leaf bud) on gene expression responses to elevational changes. To some extent, all tissues and organs may cooperate in response to environmental changes.

### Gene expression changes accompanying elevational environment adaptation

The results of gene modules related to elevation based on a gene co-expression network analysis (WGCNA) showed negative correlation coefficients between modules and altitude (r < 0) (**Figure 4**), indicating that genes in these modules were less expressed with higher altitude. We identified different environmental adaptation genes in the two organs. In the late flower bud, they were related to terpenoid metabolism (**Table 2**; **Figures 5A, C**), such as *RsHMGR* (DN1574_c1_g1) and *RsTPS* (DN13962_c0_g1, DN14863_c0_g1, etc.), which are key genes in terpenoid synthesis, while genes identified only in early leaf bud were mainly related to anthocyanin biosynthesis, leaf senescence and photosynthesis (**Table 2**; **Figures 5B, D**), such as the anthocyanin synthesis-related genes *RsCHS* (DN1880_c2_g1, DN31591_c0_g1, etc.) and *RsF3’5’H* (DN2888_c0_g1) (**Figure 6**; **Supplementary Figure S6**). The expression levels of these genes decreased with increasing elevation. Terpenes are one of three main component classes of flower scent that is one of the most important traits for plant adaptation and evolution (Knudsen et al., 2006; Raguso, 2008; Wang et al., 2021a). Anthocyanins are secondary metabolites belonging to flavonoids determine the resistance of species to extreme environments (Grotewold, 2006; Tanaka et al., 2008). Environmental factors (temperature, light quality, humidity and oxygen concentration) change along an elevational gradient, with temperature and oxygen concentration decreasing with elevation, while light intensity (UV-radiation) increases (Körner, 2007). In *Rhododendron* plants, scent-related terpenes are the main components of their floral fragrance and the main substances that attract insects (Dudareva et al., 2005; Wang et al., 2021a). Wang et al. (2021a) demonstrated that species in *Rhododendron* sect. *Azaleastrum*, mainly distributed at low elevations, have fragrant flowers, but generally exhibit light or faded colors, compared to high-elevation rhododendrons, and the expansion of terpene synthase genes (*TPSs*) in *R*. *ovatum* not only promotes floral scent production but is also related to a greater diversity of the floral fragrant compounds. However, due to the limitation of abiotic factors, pollinators are generally rare in higher elevational environments (Hargreaves et al., 2015; Tong et al., 2021). Although the successful attraction of plant floral organs to pollinators determines the reproductive success rate of a species, to a certain extent, there must be an evolutionary trade-off for plants to adapt to the environment in this long evolutionary process (Armbruster, 2014). Because more fragrance production equates to more energy consumption, while it is extremely difficult for plant biomass accumulation where induced by harsh environmental factors at high elevations (Lavorel and Grigulis, 2012), when it is only to attract insects that are relatively rare there (Totland, 2001). It is also a balance for pay (energy expenditure on floral fragrance) and gain (attraction of pollinators). Notably, our study supported the hypothesis of a contrasting living strategy that were proposed for *Trifolium repens* (Hofmann and Jahufer, 2011) and *Cyclocarya paliurus* (Du et al., 2021). This is also a manifestation of plant adaptation to the environment as a long evolutionary process (Cruden, 1972; Hargreaves et al., 2015).

The accumulated anthocyanins are secondary metabolites of flavonoids (Tanaka et al., 2008; Jaakola, 2013), usually implicated in flower color as in the variety here (Ye et al., 2021). They are also frequently produced in young shoots, including leaf buds (Lee, 2002). Leaf buds often flush red synchronously during expansion that can be a widespread and visually striking phenomenon (Karageorgou and Manetas, 2006; Alberto et al., 2011). However, the red flushing appearance of leaf buds (as indicated in **Figure 1A** (3)) in many plants is transient, and most leaves contain considerable quantities of anthocyanins only in the juvenile stages (Dominy et al., 2002; Zhang et al., 2016), and can be a response to environmental stress (Lee, 2002). Among various environmental factors that affect anthocyanin biosynthesis, light intensity (UV radiation) and temperature are particularly important (Jaakola and Hohtola, 2010; Naing and Kim, 2021). It is generally acknowledged that strong light intensity can enhance the expression of genes related to anthocyanin biosynthesis and increase anthocyanin levels (Jaakola, 2013; Zhang et al., 2018). Studies have shown that within a certain range, light intensity can activate phytochromes and promote the synthesis or activation of a series of light-regulated enzymes such as phenylalanine lyase (*PAL*), chalcone synthase (*CHS*), and flavonoid glucosidase (*UFGT*), which affect the content and proportion of anthocyanins, thereby resulting in the coloration of leaves (Jaakola and Hohtola, 2010; Dong and Lin, 2021). However, the response of plants to light intensity varies in different environmental conditions. For example, Senica et al. (2017) revealed an increased anthocyanin content with increasing elevation in leaves of elderberry (*Sambucus nigra*), and Du et al. (2021) for *Cyclocarya paliurus*. Therefore, we hypothesize that increase on anthocyanin levels may be linked to the increase in light intensity across a certain elevational range. While at extremely high-elevation environments (> 3,000 m), gene expression is inhibited and hinders the synthesis and accumulation of anthocyanin in an early leaf bud. It has been shown that foliar anthocyanins are correlated with resistance to biotic and abiotic agents such as herbivores, cold and excess radiation (Gould et al., 2000; Close and Beadle, 2003). On the one hand, observations in the field showed that leaf buds, especially at lower elevation may be susceptible to herbivore damage, for insects may show preferences for young leaves for food or oviposition (Close and Beadle, 2003). Therefore, selective pressures of insect herbivory at lower elevations could elicit increased anthocyanin production (antiherbivore hypothesis) (Kost et al., 2020), because foliar anthocyanins and some other phenolics can serve as potent deterrents against generalist herbivores (Karageorgou and Manetas, 2006; Kost et al., 2020). On the other hand, excessive light intensities at high elevations may predispose leaves to photoinhibition of photosynthesis and may also cause damage to leaf buds because of their immature photosynthetic machinery (Miranda et al., 1981). However, Karageorgou and Manetas (2006) found that leaf thickness rather than anthocyanins could appreciably reduce the risk of photo-oxidative damage in young leaves in the field, and even the photoprotective function of anthocyanin accumulations in mature leaves under stress is transient. Thus, the effect of leaf thickness on photoprotection effectiveness may have been underestimated, which generally happened at high elevation. It could thus also be hypothesized that a substrate competition exists between lignin and anthocyanin biosynthesis (Hu et al., 2021). Plants occurring in high elevations can increase lignin deposits and thickness in the cell walls by competing for common substrates under UV exposure to reduce potential excessive light damage (Kost et al., 2020). In the present study, the down-regulation of DEUs involved in the anthocyanin metabolic pathway indicates that *R*. *sanguineum* var. *haemaleum* at high elevation experience a reduction in the expression of genes related to anthocyanin accumulation. This reduction is possibly induced by selective pressures of insect herbivory from environmental changes and substrate competition in the biosynthesis pathway. Our results are in accordance with a study on *Zea mays* where lowland maize landraces showed higher expression of several confirmed and putative genes involved in anthocyanin biosynthesis as compared to highland landraces (Kost et al., 2020). In addition, a high light intensity is also accompanied by low temperatures in high elevational environments, and studies have shown that low temperatures inhibit anthocyanin synthesis (Christie et al., 1994; Naing and Kim, 2021). Choi et al. (2009) reported that anthocyanin accumulation is light dependent at low temperatures and its synthesis is down-regulated at low temperatures. Collectively, the low expression level of anthocyanin biosynthesis-related genes at higher elevations can therefore be ascribed to a combined effect of biotic and abiotic factors.

### Response strategies of flowers and leaves to high elevational environments

The adaptation of organisms to elevational environments is a complex process (Abbott and Brennan, 2014; Sun et al., 2014). In plant species, some response mechanisms and related signalling systems are excited and reinforced to adapt to different environmental stresses (Weigel and Nordborg, 2015; Hao et al., 2019). As the two most important organs of plants, those of flowers (as reproductive organs) and leaves (as primary photosynthetic organs), representing the above-ground parts of the plant, their occurrence along an elevational gradient present, to a certain extent, the species’ responsiveness to environmental variation (Frei et al., 2014; Lopez-Goldar and Agrawal, 2021). In the present study, we found that the metabolic pathways and candidate genes related to altitude identified from flowers and leaves were significantly different (**Figure 5**; **Table 2**). Genes from the late flower bud were mainly related to terpenoid metabolism, fatty acid elongation and flower organ development, while genes related to anthocyanin biosynthesis, photosynthetic antenna proteins and leaf senescence were identified in early leaf buds. Moreover, the expression of these genes was negatively correlated with altitude, that is, the higher the altitude, the lower their expression levels. When plants experience abiotic stress, they try to adapt to the stressful condition by adjusting the expression of a series of genes involved in complex networks (Nagano et al., 2019; Geng et al., 2021; Tokunaga et al., 2022).

Studies have shown that flower organ, as a very short-lived reproductive organ, mainly functions to attract pollinators, and produce seeds, while leaf organ is responsible for photosynthesis and energy supply (Bazzaz et al., 1979; Alderson and Rowland, 1995), and are long-lived in the evergreen *Rhododendron sanguineum*. Thus, it is not surprising that these two organ types behave very differently in response to changes in elevation. Terpenoids are the largest class of secondary metabolites in plants (Dudareva et al., 2005). Terpenes can be emitted from vegetative and floral organs to protect organs from heat burns produced by photosynthesis, and they can also participate in certain chemical ecological processes as signal molecules to attract insects and other pollinators (Pichersky and Gershenzon, 2002; Dudareva et al., 2004). Anthocyanins are secondary metabolites of flavonoids and play an important role in plant stress response (Tanaka et al., 2008; Du et al., 2021). The leaf is the main organ of plants exposed to air for photosynthesis, and it is more sensitive to the surrounding environment (including biotic and abiotic factors), mainly in the content of secondary metabolites (Hartmann, 1996; Landi et al., 2020). Moreover, under the long-term influence of external ecological factors, the morphological structure of leaves has great variability and plasticity, which is generally closely related to plant growth strategies and the ability to utilize resources (Obeso, 2002; Taylor et al., 2012; Rathee et al., 2021). Consequently, there are obvious functional divisions and constraints between the two organs from an ecological point of view.

## Conclusion

In the present study, comparative analysis of transcriptome data on late flower and early leaf bud at four different elevations of *R*. *sanguineum* var. *haemaleum* showed that the overall gene expression patterns clustered independently according to the different organs used. Different environmental adaptation genes identified in the floral and leaf organs are mainly related to terpenoid metabolism (*RsHMGR*, *RsTPS*) and anthocyanin biosynthesis (*RsCHS*, *RsF3’5’H*), respectively. The expression levels of these genes decreased with increasing elevation, which also determined the type of secondary metabolite to some extent. The heterogeneous environment resulting from the elevational change may be the main factor affecting gene expression, which illustrated that plant species may adopt varying adaptive strategies to cope with environmental stresses. However, the response of plants to the environment is a complex and comprehensive process, and it is also the result of the coordination and interaction of various traits. Thus, further study on the determinants of secondary metabolite content and quantification of environmental variables across elevations should be investigated to precisely elucidate the plant’s adaptability. Overall, this study provides new insights into the molecular mechanisms of an alpine species’ response to heterogeneous environment caused by elevation, and will shed insights into further studies on high elevational adaptation among rhododendrons and other alpine species in general.

## Data availability statement

The data presented in the study are deposited in the National Center for Biotechnology Information (NCBI) Sequence Read Archive (SRA) repository. The names of the repository/repositories and accession number(s) can be found below: http://www.ncbi.nlm.nih.gov/bioproject/PRJNA916369.

## Author contributions

LG and DL conceived and designed the research. LY, YL, JZ and WZ carried out field work and sample collection. LY performed the experiments and analyzed the transcriptomic data with assistance from JZ. LY wrote the original draft with critical input from MM and LG. All authors contributed to the article and approved the submitted version.
