# Supplementary material for: Variation in gene expression along an elevation gradient of Rhododendron sanguineum var. haemaleum assessed in a comparative transcriptomic analysis
Source: Front Plant Sci. 2023 Mar 21;14:1133065. doi: 10.3389/fpls.2023.1133065 (PMC10070981; doi:10.3389/fpls.2023.1133065)
Supplement: Supplementary file 1 [file DataSheet_1.docx]

**Supplementary Information**

**Variation in Gene Expression along an Elevation Gradient of *Rhododendron*** ***sanguineum* var. *haemaleum* Assessed in a Comparative Transcriptomic Analysis**

Linjiang Ye^1,2,3^, Michael Mӧller^4^, Yahuang Luo^1,5^, Jiayun Zou^1^, Wei Zheng^1,6^, Jie Liu^1^, Dezhu Li^3,5,6^, Lianming Gao^1,5*^

^1^ CAS Key Laboratory for Plant Diversity and Biogeography of East Asia, Kunming Institute of Botany, Chinese Academy of Sciences, Kunming 650201, Yunnan, China;

^2^ Key Laboratory of Plant Resources and Biodiversity of Jiangxi Province, Jingdezhen University, Jingdezhen 333000, Jiangxi, China;

^3^ Germplasm Bank of Wild Species, Kunming Institute of Botany, Chinese Academy of Sciences, Kunming 650201, Yunnan, China;

^4^ Royal Botanic Garden Edinburgh, Edinburgh EH3 5LR, Scotland, United Kingdom;

^5^ Lijiang Forest Biodiversity National Observation and Research Station, Kunming Institute of Botany, Chinese Academy of Sciences, Lijiang 674100, Yunnan, China;

^6^ University of Chinese Academy of Sciences, Beijing 10049, China.

**Table supporting information**

**Table S1** Basic information of transcriptome sequencing and alignment rates of the 24 samples of *Rhododendron* *sanguineum* var. *haemaleum*.

| **sample name** | **clean reads** | **clean bases (bp)** | **Q30** | **GC content** | **Mapping rates** |
| --- | --- | --- | --- | --- | --- |
| F_RsH3000_01 | 54,680,282 | 8,202,042,300 | 92.95% | 48.61% | 95.66% |
| F_RsH3000_02 | 43,226,658 | 6,483,998,700 | 90.28% | 48.84% | 94.27% |
| F_RsH3000_03 | 42,867,532 | 6,430,129,800 | 90.81% | 48.19% | 94.91% |
| F_RsH3200_01 | 57,554,512 | 8,633,176,800 | 92.97% | 48.51% | 95.82% |
| F_RsH3200_02 | 43,351,928 | 6,502,789,200 | 91.46% | 48.02% | 95.39% |
| F_RsH3200_03 | 50,608,754 | 7,591,313,100 | 91.32% | 47.86% | 94.27% |
| F_RsH3600_01 | 51,196,206 | 7,679,430,900 | 93.01% | 48.30% | 96.07% |
| F_RsH3600_02 | 52,206,348 | 7,830,952,200 | 89.22% | 48.41% | 94.83% |
| F_RsH3600_03 | 45,658,874 | 6,848,831,100 | 90.67% | 48.21% | 95.35% |
| F_RsH3800_01 | 54,988,490 | 8,248,273,500 | 92.92% | 48.55% | 95.78% |
| F_RsH3800_02 | 47,585,482 | 7,137,822,300 | 90.95% | 47.28% | 94.09% |
| F_RsH3800_03 | 48,544,918 | 7,281,737,700 | 89.75% | 48.20% | 94.40% |
| L_RsH3000_01 | 52,646,696 | 7,897,004,400 | 93.63% | 47.69% | 95.81% |
| L_RsH3000_02 | 50,444,294 | 7,566,644,100 | 91.92% | 48.54% | 95.76% |
| L_RsH3000_03 | 43,181,462 | 6,477,219,300 | 90.94% | 46.89% | 95.03% |
| L_RsH3200_01 | 59,403,462 | 8,910,519,300 | 91.16% | 48.81% | 95.73% |
| L_RsH3200_02 | 40,622,598 | 6,093,389,700 | 92.03% | 48.06% | 95.68% |
| L_RsH3200_03 | 42,281,166 | 6,342,174,900 | 91.64% | 48.39% | 95.84% |
| L_RsH3600_01 | 53,440,592 | 8,016,088,800 | 93.47% | 46.35% | 95.96% |
| L_RsH3600_02 | 41,239,386 | 6,185,907,900 | 90.34% | 46.80% | 94.23% |
| L_RsH3600_03 | 44,061,042 | 6,609,156,300 | 91.02% | 46.84% | 94.96% |
| L_RsH3800_01 | 41,698,868 | 6,254,830,200 | 93.82% | 46.83% | 95.55% |
| L_RsH3800_02 | 49,780,986 | 7,467,147,900 | 91.09% | 46.83% | 94.45% |
| L_RsH3800_03 | 51,298,928 | 7,694,839,200 | 89.32% | 47.47% | 93.85% |

**Note:** F – RNA-seq libraries from floral bud; L – RNA-seq libraries from leaf bud; RsH – *R. sanguineum* var. *haemaleum*; Q30 – phred score that the base call accuracy of 99.9%; 01-03 – biological replicates.

**Figure supporting information**

**
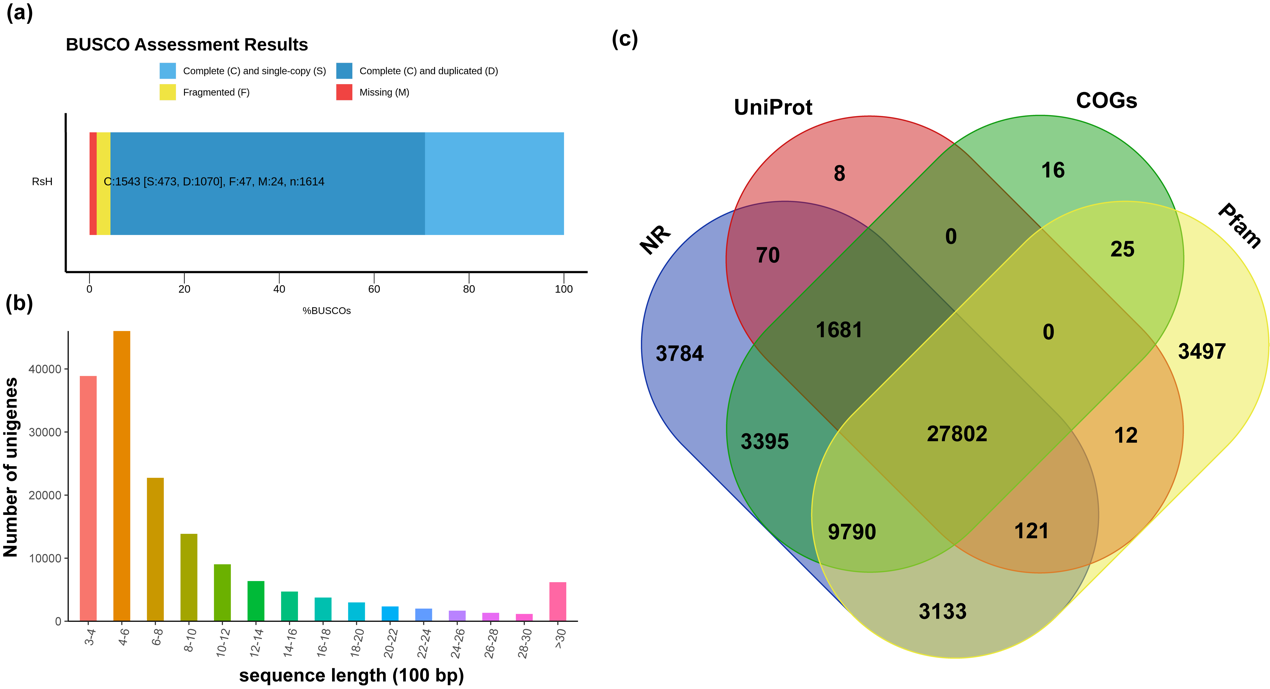
**

**Figure S1** Busco assessment, sequence length distribution and venn diagram of the assembled unigenes BLAST searches against four public databases, including NR, UniProt, COGs and Pfam. The numbers of unigenes that have significant hits against the four databases are shown in each intersection of the venn diagram.

**
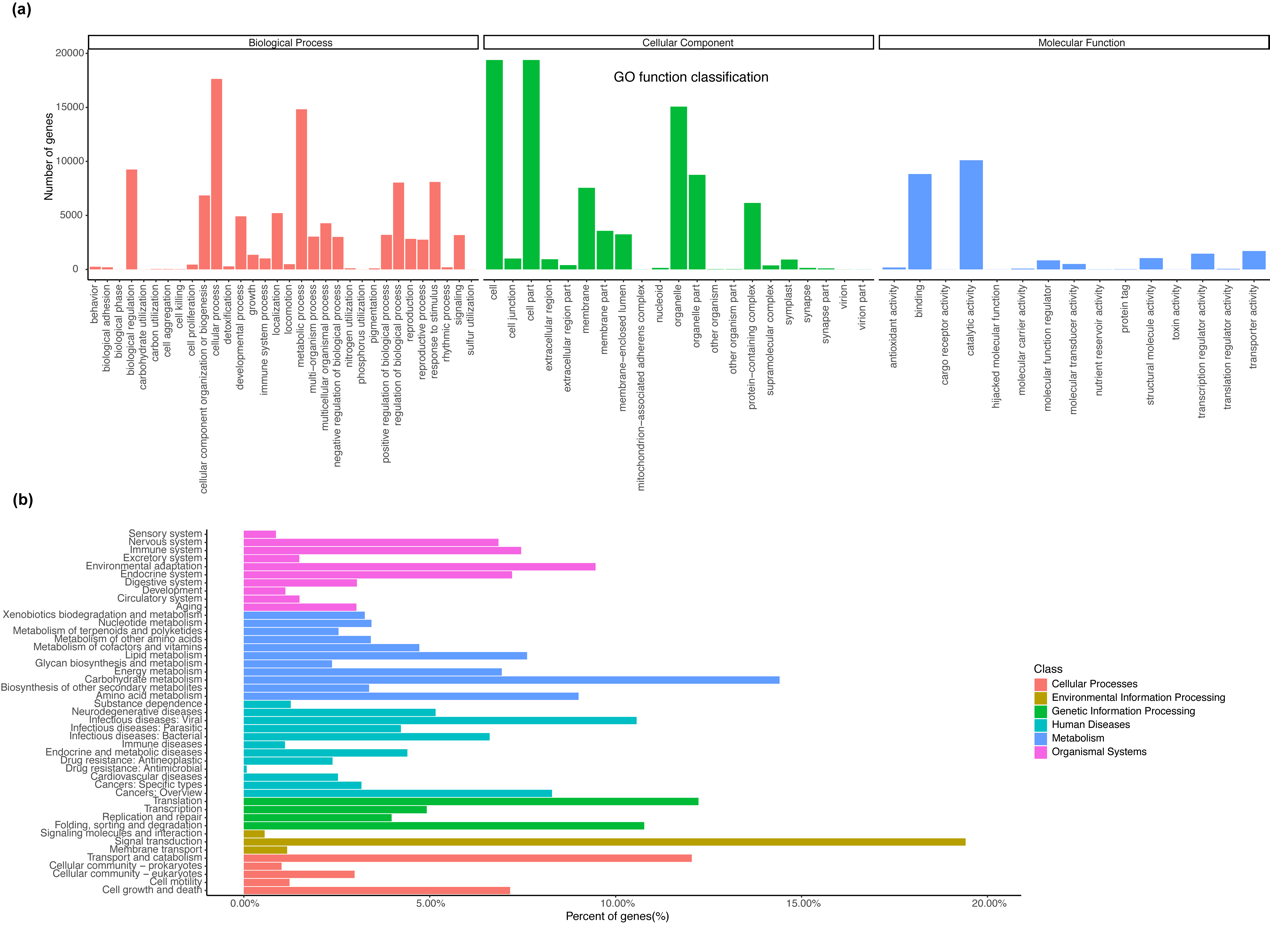
Figure S2** Annotation of the unigenes from *R. sanguineum* var. *haemaleum* searching against different databases. (a) Gene Ontology (GO) functional classification (the x-axis indicates GO classification, and the y-axis indicates the number of unigenes in the GO databases). (b) KEGG metabolic pathway (the x-axis is the ratio of the number of genes, and the y-axis is the name of the KEGG metabolic pathway).

**
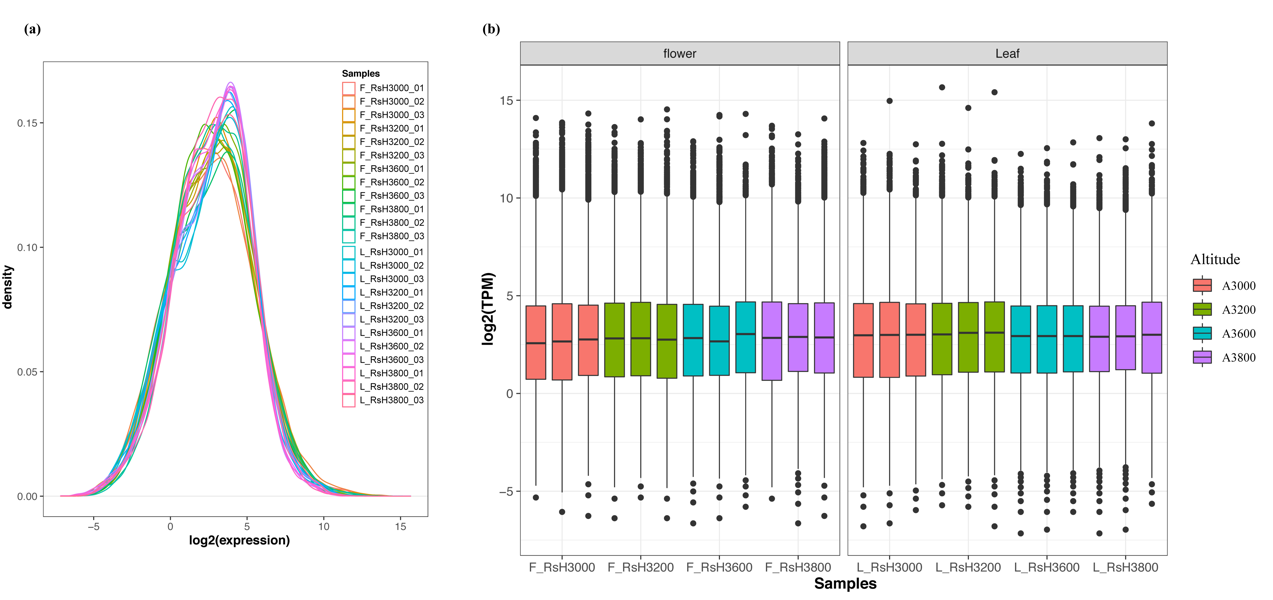
Figure S3** Gene expression density (a) and distribution (b) of 24 individual libraries of late flower buds and early leaf buds.

**
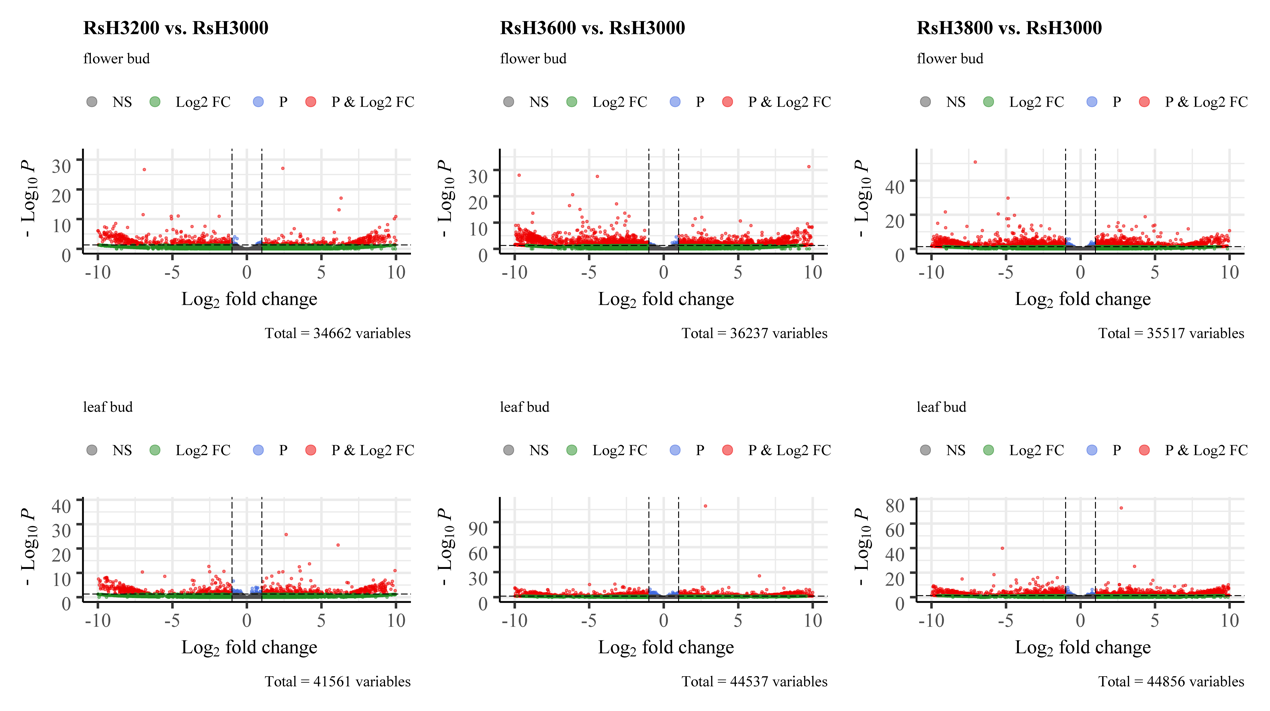
**

**Figure S4** Volcano map of differentially expressed genes among three comparison groups in flower bud and leaf bud, respectively.

**
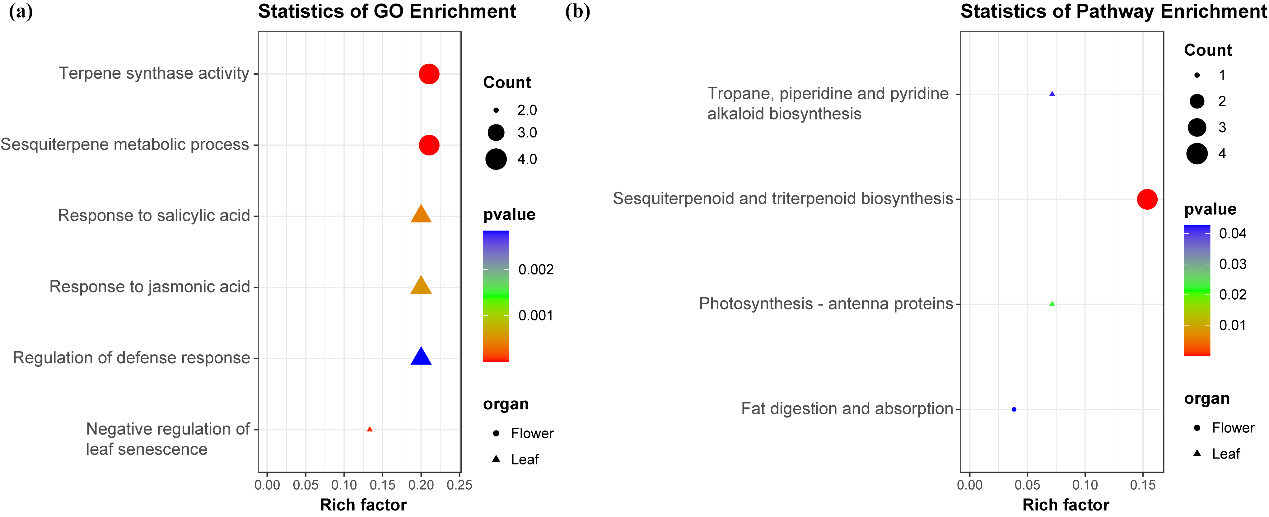
Figure S5** GO enrichment (a) and KEGG enrichment (b) of the significant differentially expressed genes (DEGs) shared in the three comparisons under different organs (late flower bud and early leaf bud).

**
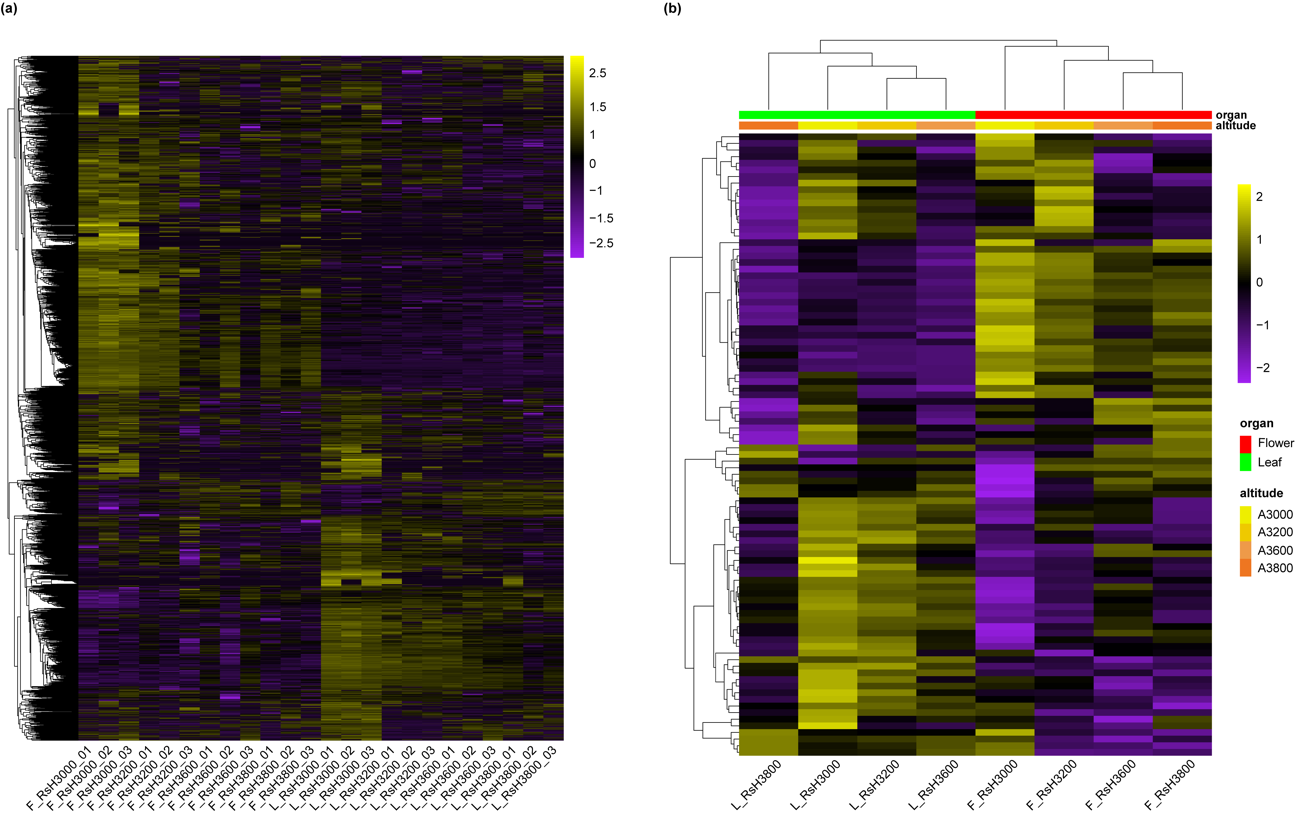
**

**Figure S6** The expression pattern of the genes identified from differently significant modules (a), and the expression heatmap of the representative genes associated with altitude (b). The expression values of each altitude were indicated by the mean of three replicates. Abbreviation: F, late flower buds; L, early leaf buds.
